# Riding the wave of success: revamping the Nourishing Networks education program

**DOI:** 10.1186/2050-2974-3-S1-O16

**Published:** 2015-11-23

**Authors:** Leanne Brown, Deanne Harris, Miriam Grotowski, Fiona Little

**Affiliations:** 1 University of Newcastle Department of Rural Health, Australia; 2 Hunter New England Health Local Health District, Australia; 3 Smith Street Practice, Australia

## 

Nourishing Networks (NN) is an innovative interprofessional learning program delivered in 2009 and again in 2014 for health professionals managing eating disorder clients in rural NSW. It was designed to enhance local networks and improve the knowledge, skills and attitudes of health professionals working with eating disorder clients.

Impetus for re-delivering the program was high turnover of staff in rural communities and need to increase awareness of existing services. Identification of clients late in the course of illness remained a reoccurring issue.

The original program consisted of a 10 week self-directed learning program with videoconferencing and a one-off workshop for health professionals. The revised program utilised a travelling roadshow to three rural locations to provide better access for outlying sites. This provided increased opportunities for networking and upskilling to a wider audience. The revised program attracted a greater range of health professionals with further involvement of general practitioners. Overall the program trained 254 participants, from nine different health professional backgrounds.

Nourishing Networks has led to improvements in clinician confidence in identifying and assessing eating disorder clients, improved quality of referrals to existing clinicians, new services in some rural communities and an increase in GPs prepared to manage the clients.

